# Legal Notes for Institutional Workers

**Published:** 1912-07-06

**Authors:** 


### LEGAL NOTES FOR INSTITUTIONAL WORKERS.

(The Editor will be pleased to answer Legal queries in this column.)

### Diseases and the Compensation Act.

Although but few of the ills to which the workers' flesh is heir are classed as "industrial" within the meaning of the Workmen's Compensation Act, there are many complaints for which compensation is awarded. The question is, Was this disease caused or aggravated by an accident to the workman arising out of and in the course of his employment? Although the County Court judge is at liberty to solve the problem himself, there is an increasing tendency to pray the services of a medical referee in aid. Indeed, we know of one judge in South Wales who invariably summons a referee in every case which has to be decided on medical evidence. Having regard to the large sums at stake we cannot but think that this practice enures to the advantage of all parties. At any rate it tends to economy, for costs mount up to an extraordinary extent when a case is contested and fought out in court.

### The Limits of Fair Comment.

A recent libel action in which the *British Medical Journal* was somewhat seriously involved draws attention to what are the metes and bounds of fair comment. It is an axiom of law that "fair comment upon a matter of public interest is no libel." The Judge having ruled upon the question whether a matter is one of public interest, it is for the jury to say whether the comment is fair or not. Criticism or comment may be severe, but it may still be fair; but it must be upon existing, and not upon suppositious facts. As was said in an old case, "That a fair and bona-fide comment on a matter of public interest is an excuse of what would otherwise be a defamatory publication is admitted. The very statement, however, of this rule assumes the matters of fact commented on to be somehow or other ascertained. It does not mean that a man may invent facts and comment on the facts so invented in what would be a fair and bona-fide manner on the supposition that the facts were true. . . . If the facts as a comment upon which the publication is sought to be excused do not exist, the foundation of the plea fails." The reason why fair comment is no libel is not difficult to explain or to understand. There must be no monopoly of opinion. If A. thinks a particular treatment is useless, or a particular medicine is wholly valueless for a certain disease, he is entitled to say so, and to say so in strong terms. The treatment and the medicine being matters of public notoriety other people are at liberty to form their own conclusions about them. Having considered the treatment, they may think that A.'s judgment in the matter need not be seriously considered. Your critic, however, must not be malicious. He may criticise a man's work, but not the man himself. If the jury come to the conclusion that what is ostensibly an attack upon a treatment is in fact a serious charge against the inventor of the treatment, they will infer a malicious or improper motive and damages will be the result.

### Compensation for Smallpox.

Although smallpox is not a disease scheduled to the Act, it may happen that a man becomes infected in such circumstances that the court will hold that the onset of the complaint was "accidental" in the legal sense. In a case recently heard at Folkestone a woman claimed compensation in respect of the death of her husband. It appeared that he had assisted to place in its coffin the corpse of a man who had died of smallpox. It was alleged, in defence, that there was no positive evidence that the germs of the disease were caught in this way; but it was proved that three men who had assisted at the burial also contracted smallpox. After this evidence the judge made an award of compensation.

### Diabetes in a Miner.

In a case recently heard at Hanley the judge had to consider the following facts. A timberer who was employed in a mine received an internal injury while at work. Compensation was paid for a considerable time, when the employers sought to have it discontinued on the ground that the man was able to resume work. It then transpired that the man was suffering from diabetes. A doctor called on his behalf said that the most eminent authorities gave injury as the principal immediate cause of the complaint. The absence of albumen in the urine went to show that the present condition was more likely to be due to accident than to long-standing prior disease. He also said that diabetes was more frequent in stout people than in thin people, the Hebrews being notoriously prone to it. The Americans were markedly free from the disease, and they were well known to be a thin race. The judge came to the conclusion that the disease was more likely to be due to accident than to natural causes, and he therefore directed that compensation should continue.

### Epilepsy: Compensation Claim Dismissed.

In a case at Salford it appeared that in December last a man of thirty-four was thrown from a lorry, sustaining injuries to his leg. He was in hospital for a week; and on February 9, 1912, was re-admitted in a state of unconsciousness due to epilepsy. After suffering from several fits he died ten days later. A doctor called on behalf of the widow, who claimed compensation, said that it was very rare for a man of this age to have epileptic fits without any apparent cause, and there was one form of the disease which might have been induced by injury. Again, in the fall an injury to the head might have been a cause. A doctor called on behalf of the employers said that if injury had caused the disease there would have been meningitis, or else some proof that germs had entered the blood through the wound. There being no evidence he came to the conclusion that the epilepsy had nothing to do with the accident. The judge found for the employers.

